# Rheologic controls on the depth dependence of megathrust earthquakes

**DOI:** 10.1073/pnas.2535447123

**Published:** 2026-06-25

**Authors:** Melodie E. French, Jonathan R. Delph, Cailey B. Condit

**Affiliations:** ^a^ Department of Earth, Environmental and Planetary Sciences, Rice University, Houston, TX 77005; ^b^ Department of Earth, Atmospheric, and Planetary Sciences, Purdue University, West Lafayette, IN 47907; ^c^ Department of Earth and Space Sciences, University of Washington, Seattle, WA 98195

**Keywords:** earthquakes, rheology, subduction, megathrust

## Abstract

The various slip behaviors of the subduction megathrust fault, including deadly megathrust earthquakes, are depth-dependent. Yet, we do not know what causes this depth dependence, in part due to variability between subduction zone thermal structures and lithological inputs. Here, we investigate controls on the nucleation depths of great earthquakes (Mw ≳ 8) and the deeper extent of seismogenesis. To do so, we create rheologic strength envelopes for six subduction zones using published constitutive relations for subducting lithologies and regional thermal models. We then compare the strength envelopes to local plate interface earthquake depths. Our synthesis shows that subducted sediments undergo a transition from frictional to predominantly viscous deformation near 300 ± 60 ^°^C a transition that correlates with the maximum nucleation depths of great earthquakes at 20 to 30 km. However, smaller plate interface earthquakes (5≤ Mw < 8) continue to nucleate below this depth, and the maximum earthquake size decreases until the base of the seismogenic zone at 40 to 60 km depth and ∼500 °C. We evaluate potential causes of seismicity below the onset of viscous deformation in these now metasediments, and conclude that the presence of lithologic heterogeneities that are loaded to frictional failure by viscously deforming metasediments is most plausible. Thus, we propose that great earthquakes can nucleate and grow into large events where all lithologies are frictional. However, the base of the seismogenic zone is deeper than the onset of viscous deformation in metasediments, which limits the growth of earthquakes into large events, and here seismicity reflects the existence and size of heterogeneity along the plate interface.

## Significance

Earth’s largest earthquakes occur along the subduction megathrust, yet there is no consensus on what physical properties control the size of the seismogenic zone or its downdip segmentation. We find that the growth of earthquakes into large events is limited to a shallower region where all rocks deform by frictional failure. Below this depth, frictional failure of the sediments is suppressed in favor of viscous creep. Smaller earthquakes continue to occur deeper though they cannot grow into large events. These earthquakes decrease in size with depth, and we propose they reflect the size of physical heterogeneity. These constraints will allow us to more accurately evaluate seismic hazards by modeling the rheology of the megathrust and the nature of its heterogeneity.

**Fig. 1.**
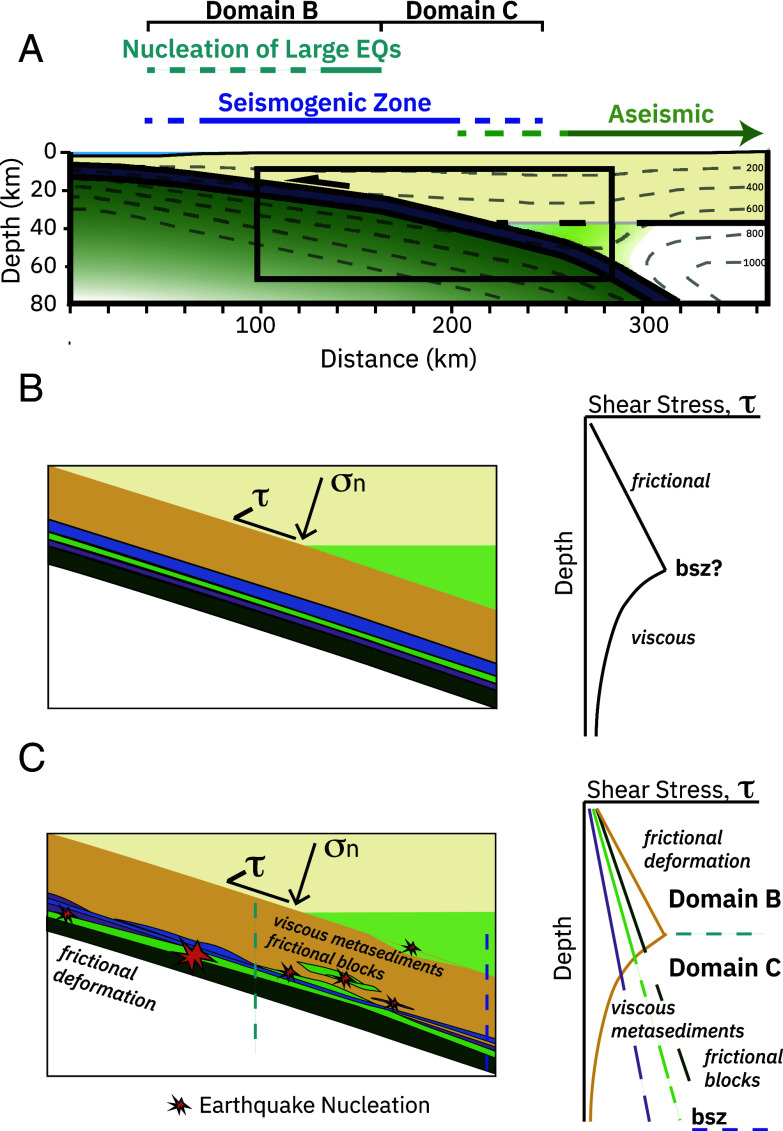
(*A*) Schematic cross-section of a subduction zone showing the general region in which the nucleation depth of large earthquakes and the base of the seismogenic zone (bsz) occur. Domains B and C are defined by ref. 1 as the depth range of great earthquake centroids and a deeper region of the seismogenic zone where smaller earthquakes nucleate and great earthquakes may slip, respectively. The black box shows the regions illustrated in (*B* and *C*). (*B*) The conceptual model we evaluate to determine whether the depths at which viscous deformation in megathrust lithologies can accommodate subduction correlate with the deepest extent at which large earthquakes nucleate or the base of the seismogenic zone. Orientations of shear (τ) and normal ($\sigma_{n}$) stresses are shown. (*C*) Our interpretation that shows a rheologic transition from predominantly frictional failure of all lithologies to viscous shearing of metasediments. Earthquakes nucleate and grow to large events above this transition, but smaller megathrust earthquakes can still nucleate without growing below this transition when blocks (e.g., metasomatic phases) are loaded to frictional failure by the shearing metasediments. These blocks can also support enough stress to propagate great earthquakes that nucleate in Domain B.

Not only do subduction megathrusts host Earth’s largest earthquakes, but they exhibit depth-dependent patterns in both slip modes and earthquake sizes. In particular, the nucleation of great earthquakes (Mw ≳ 8) occurs over a shallower depth range (<∼35 km, Domain B of ref. 1) than small to moderate earthquakes (∼35 to 60 km, Domain C of ref. 1) (Fig. 1*A*). While broad correlations have been drawn between earthquake nucleation depth, or the “seismogenic zone,” and physical conditions like temperature and fluid pressure, we do not know what processes control the depth extent of the seismogenic zone, which we refer to as the “base of the seismogenic zone,” or segmentation of the seismogenic zone (2, 3). This knowledge gap is especially notable compared to continental and oceanic crustal faults, where large earthquakes do nucleate at depths near the base of the seismogenic zone, and this depth can be explained using the rheology of simple, monomineralic lithologies (4–6). Although great earthquakes nucleate over a shallower depth range in subduction zones, they can slip to depths closer to the base of the seismogenic zone (i.e., into Domain C of ref. 1); thus, understanding the causes of segmentation will lead to better constraints on seismic hazards.

Although the base of the megathrust seismogenic zone is often approximated at ∼350 to 450 °C (7–9), this broad range of temperatures makes it challenging to identify the mechanisms that cause depth-dependent behavior. Recent studies seek to explain different aspects of the seismogenic zone, but do not explain depth-dependent segmentation. For instance, the onset of pressure solution creep in sediments is proposed to control the base of the seismogenic zone in Cascadia (10), but their seismicity rates are too low to relate this process to depth-dependent earthquake distributions. The model of refs. 11 and 12 relates the depth of great earthquake slip to the proximity and physical properties of serpentine in the mantle wedge, and also explains smaller earthquakes that define the base of the seismogenic zone as distributed serpentine heterogeneity. However, this model does not explain the physical properties that limit the nucleation depth of great earthquakes, nor take into account lithologic (and rheological) heterogeneity.

Our understanding of what controls earthquake depths along the megathrust is hindered by significant variability between subduction zones, making the synthesis of behaviors difficult to untangle. For instance, thermal structures and gradients vary significantly between subduction zones, and each plate interface also comprises a complex mix of lithologies with variable rheologies including (meta)sedimentary, ultramafic, mafic, and metasomatic rocks (13–16) which often have heterogeneous block-in-matrix structures (e.g., ref. 17). Inputs of sediment compositions and thicknesses also vary across margins. Consequently, the lithologies involved in slip and how their strengths change with depth and strain rate remain uncertain. Despite this variability, the segmentation of fault slip into depth-dependent domains is consistent to first order across subduction zones (1), but the rheological controls have not yet been fully explored.

**Fig. 2.**
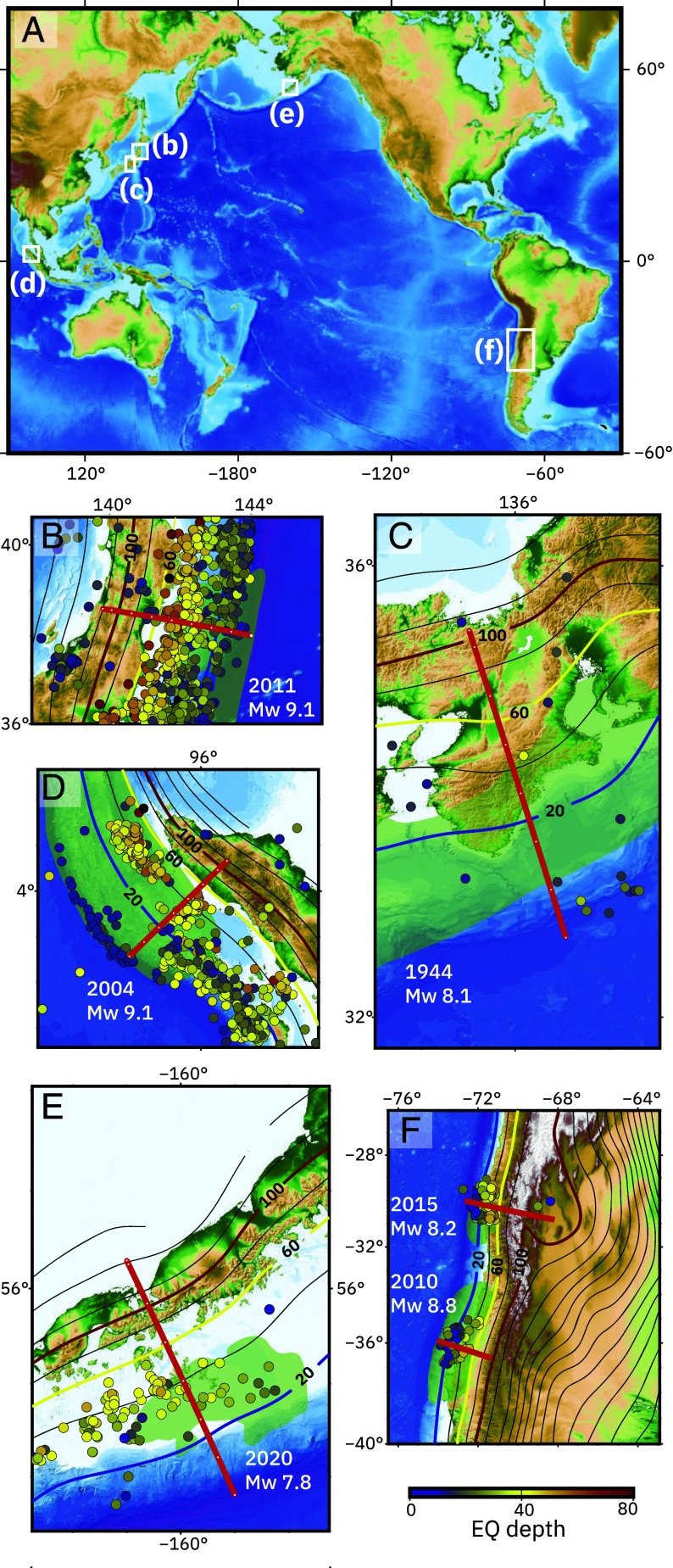
(*A*) Map showing the locations of the six subduction segments for which we create strength profiles to compare with local seismicity (*SI Appendix*, Figs. S3–S7). (*B*–*F*) Map projections of Mw ≥ 5 thrust events are shown. Green patches are regions of slip from large earthquakes. Red lines indicate the location of the profiles studied. Slab contours are shown in 20 km intervals and are from Slab 2.0 (20). (*B*) Honshu with approximate slip region of the 2011 Mw 9.1 earthquake (21), (*C*) Nankai with the slip region for the 1944 Mw 8.8 earthquake (22), (*D*) Sumatra with the slip region of the 2004 Mw 9.1 earthquake (23, 24), (*E*) the Shumagin segment of the Aleutians with the slip region of the 2020 Mw 7.8 earthquake (25), (*F*) central Chile and south-central Chile with the slip region of the 2010 Mw 8.8 Maule earthquake (26) and the 2015 Mw 8.0 Illapel earthquake (27).

When deformation is localized to a single lithology, the maximum possible extent of seismogenesis is the depth at which thermal weakening allows the rock to flow viscously at a lower stress than required for frictional failure (although it is often slightly shallower and controlled by rate-and state frictional properties) (Fig. 1*B*) (18, 19). Along the subduction megathrust, this transition from frictional failure to distributed viscous flow will occur at different temperatures, and hence depths, in different lithologies which have differing rheologies. In this study, we relate depth-dependent changes in seismicity to the depths at which each lithology may accommodate subduction by viscous processes (Fig. 1*B*). To do so, we first create temperature profiles for six subduction segments, Honshu, Nankai, Sumatra, central Chile, south-central Chile, and the Shumagin segment of the Aleutians (Fig. 2 and Table 1), using the thermal model of ref. 30. These segments were selected because they encompass a range of thermal gradients, are all dominated by siliciclastic sediment input, and have all experienced instrumentally recorded very large to great earthquakes. Next, we create strength profiles for each of these segments. We take the shear strength for frictional failure to be controlled by an effective frictional coefficient, $\mu^{\prime }$, and we assess the depth at which the shear strength for viscous deformation of metasediments, serpentinite, and blueschist (typical subduction lithologies) can accommodate plate boundary deformation at lower stress than frictional failure (Fig. 1*B*). Uncertainty in this depth is quantified using Monte Carlo simulations that consider uncertainties in temperature gradient, $\mu^{\prime }$, and layer thickness. We then compare strength profiles to the depths of megathrust earthquakes to assess which rheologic transitions correlate with the nucleation depth of large ruptures and the base of the seismogenic zone, which we identify as the deepest nucleation of Mw ≥ 5 megathrust earthquakes.

**Table 1.** Earthquake and rheologic conditions

	BSZ (km)	BSZ temp (°C)	BSZ (28) (km)	F-V (median) (km)	F-V (16 to 84%) (km)	F-V temp (°C)	MWC (km)	Large EQ (km)
Honshu	61	520 ± 200	60	39	33 to 50	300 ± 60	30	29
Nankai	40	450 ± 170	35	27	20 to 38	330 ± 60	30	25
Sumatra	57	450 ± 170	53	36	29 to 47	290 ± 60	30	29
Shumagin	49	450 ± 170	55	36	30 to 47	320 ± 60	33	28
Central Chile	57	480 ± 180	51	36	30 to 47	290 ± 60	35	29
South-central Chile	51	500 ± 190	51	31	26 to 41	290 ± 50	35	23

BSZ: Base of the seismogenic zone (results from this study are in the second column and from ref. 28 are in the fourth column for comparison), BSZ Temp determined from thermal models with up to 40% uncertainty (29), friction-to-viscous transition, MWC: Mantle Wedge Corner, Large EQ: Hypocenter depth of large earthquakes.

## Results

**Fig. 3.**
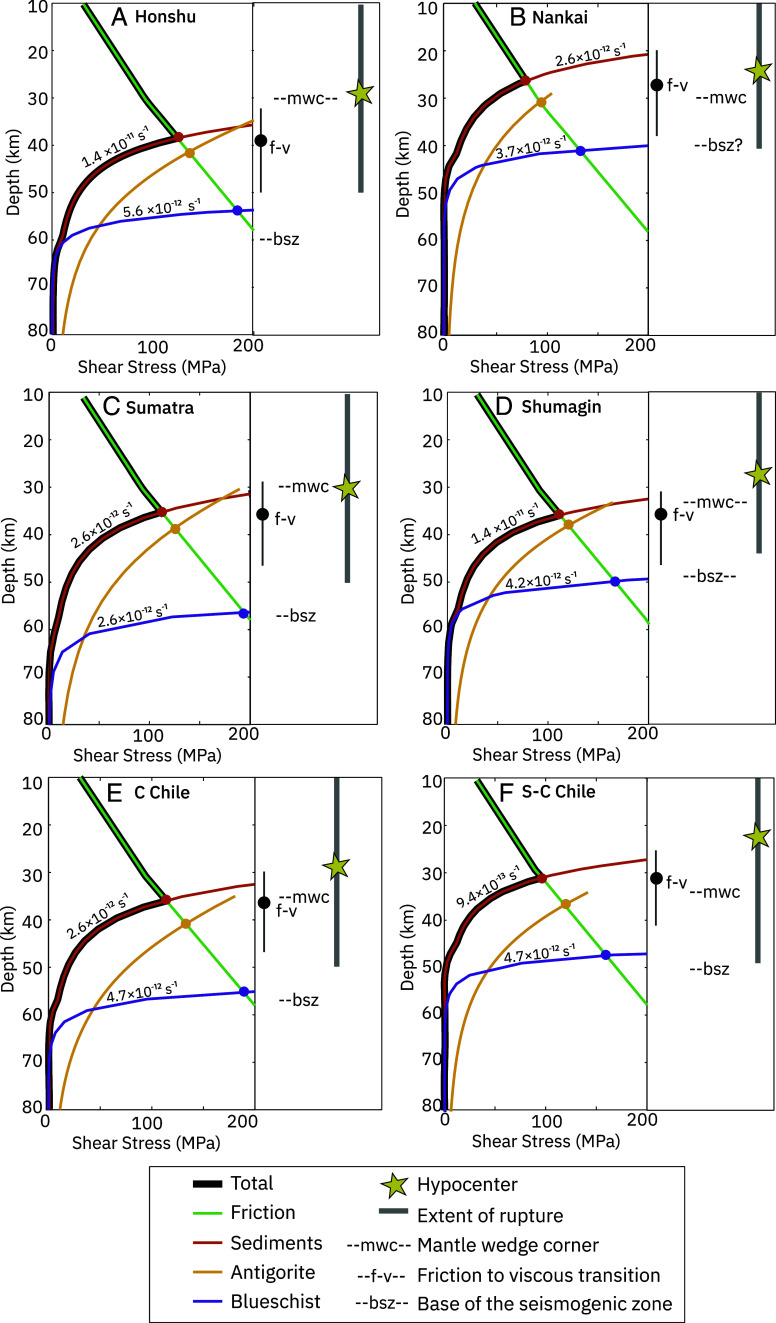
Example megathrust strength profiles and constraints on slip behavior for the six subduction segments. The viscous strengths of sediments, antigorite-rich serpentinite (beginning at the mantle wedge corner, mwc), and glaucophane in blueschist are shown. In these plots the effective friction coefficient, $\mu^{\prime }$, is 0.12, the mean value evaluated, and strain rates for viscous deformation are plate velocities divided by sediment thickness (*SI Appendix*, Table S1) or 500 m (antigorite and blueschist) and are shown. Dots indicate the depth at which viscous deformation of each lithology accommodates plate boundary deformation. The frictional-to-viscous transition of metasediments is indicated with a black dot and bar that shows the median and 16 to 84% percentile ranges from Monte Carlo simulations of uncertainties in temperature, frictional failure strength, and sediment thickness (*SI Appendix*). Stars and gray bars represent the hypocenter and the depth range that slipped during recent large or great earthquakes along each margin. The base of the seismogenic zone (bsz), defined by the depth extent of nucleation for Mw ≥ 5 earthquakes, is shown. (*A*) Honshu, Japan (*SI Appendix*, Fig. S5). 2011 Mw 9.1 Tokohu earthquake (31). (*B*) Nankai (*SI Appendix*, Fig. S6). 1944 Tonanaki earthquake (22, 32). (*C*) Sumatra (*SI Appendix*, Fig. S7). 2004 Mw 9.1 Sumatra-Andaman earthquake (23, 24). (*D*) Shumagin (*SI Appendix*, Fig. S8). 2020 Mw 7.8 earthquake (33, 34). (*E*) Central Chile. 2015 Mw 8.3 Illapel earthquake (27) (*SI Appendix*, Fig. S9). (*F*) South-central Chile. 2010 Mw 8.8 Maule earthquake (26, 35) (*SI Appendix*, Fig. S9).

Despite differences in thermal gradient, subducted sediment thickness, and seismicity at different margins, we see correlations between predicted rheology and earthquake nucleation patterns (Fig. 3) (36). Our results show that the metasediments are the first lithology to accommodate subduction through viscous deformation, and that this happens near 300 ± 60 ^°^C. Furthermore, even considering significant uncertainties and our conservative model of metasediment rheology, this transition to viscous accommodated deformation is within the seismogenic zone of all margins. However, none of the large earthquakes nucleated below this depth, which we refer to as the “frictional-to-viscous” transition. At all margins, the base of the seismogenic zone continues to greater depths, extending below the mantle wedge until temperatures near 500 °C (Table 1)

Interestingly, the base of the seismogenic zone is near the predicted depth at which frictional failure is suppressed in favor of viscous deformation in blueschist and potentially also antigorite serpentine, depending on the effective friction coefficient and the thicknesses of deforming layers (Fig. 3 and *SI Appendix*, Fig. S2). For instance, in Fig. 3 we assume a shear zone thickness of 500 m and $\mu^{\prime }$ of 0.12, and the base of the seismogenic zone is deeper than the predicted suppression of frictional failure in antigorite. However, when we evaluate a range of possible $\mu^{\prime }$ (*SI Appendix*, Fig. S1) or thicknesses (*SI Appendix*, Fig. S2), we find that there may be a correlation between the suppression of frictional failure in antigorite and the base of the seismogenic zone.

## Discussion

### Depth-Dependent Segmentation.

At shallow depths, before viscous deformation of the metasediments accommodates subduction slip rates, all lithologies are expected to fail frictionally (Fig. 3). This is consistent with field observations that show that brittle faults are hosted within both the sediments and metabasalts (37–42). Because the base of this frictional regime is consistently near or below the depth of large earthquake nucleation (Fig. 3), we propose that large earthquakes tend to nucleate within this region, consistent with Domain B of ref. 1. This is not because their nucleation stage is necessarily distinct, but because despite the presence of heterogeneity, small earthquakes can grow into large events when all the surrounding rock is near frictional failure and can experience dynamic weakening, as most lithologies do (43) (Fig. 1 *A* and *C*). However, once these events are of sufficient size, they can also propagate downdip for significant distance before arresting, even into the region in which the sediments are deforming viscously.

Small to moderate earthquakes nucleate below the frictional-to-viscous transition, indicating that even as the metasediments flow, frictional failure of other lithologies continues. This depth range correlates with Domain C of ref. 1 (Fig. 1*A*). We propose that structural and mechanical heterogeneity is the best explanation for seismogenesis in this domain (Fig. 1*C*). Often heterogeneity is envisioned in 2-dimensions as frictionally unstable patches surrounded by frictionally conditionally stable fault rock (1, 3, 44), and we propose some clarity to this conceptual model; namely, heterogeneity exists as large blocks within and adjacent to the viscously shearing metasediments, and that the block lithologies fail by seismogenic slip (e.g., km-scale block-in-matrix structures). In this case, we propose that viscous shearing of the metasediments enhances stresses in the blocks that drive frictional failure of isolated patches (e.g., refs. 39, 45, and 46). However, unlike in Domain B, viscous deformation is strongly rate-strengthening and also keeps the surrounding stress low; this will promote the arrest of small to moderate ruptures that nucleate. Thus, we propose that in Domain C, smaller earthquakes cannot propagate to become large (or great) because viscously deforming metasediments are a strong barrier to rupture propagation.

**Fig. 4.**
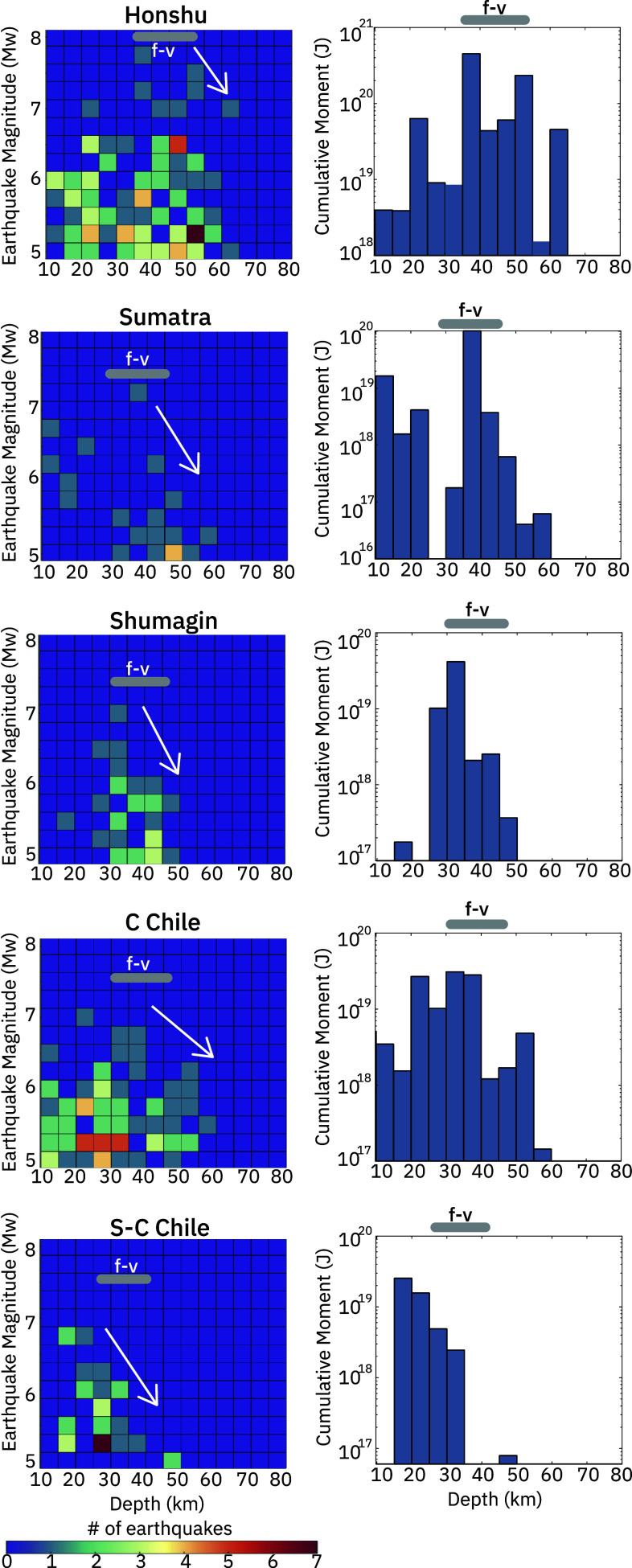
(*Left*) Heat maps showing the number of megathrust earthquakes of given magnitudes (Mw) and their hypocentral depths. (*Right*) Histograms show the sum of the seismic moments within a given nucleation depth range (e.g., cumulative seismic moment within a histogram bin). Earthquakes are thrust events within 75 km of each transect along strike and within 7 km orthogonal distance to the slab. Moment tensors are from the Global CMT Catalog (49, 50) and depths are from the ISC Bulletin (51, 52). White arrows indicate decreasing maximum magnitude with depth. The frictional-to-viscous transition is indicated as “f-v.” Earthquakes greater than Mw 7.8 are not included in the heat maps or histograms.

The rupture areas of Mw ≥ 5.0 events are ≥10 km^2^ with widths ≥2 km (47), suggesting km-scale heterogeneity is necessary to explain the observations. This is consistent with geologic observations showing km-scale lenses of mafic and ultramafic rock within metasediments (48). We can use the magnitudes of earthquakes at these depths to provide constraints on the size distribution of lithologic heterogeneity. We plot the number of small to moderate earthquakes of a given magnitude (<7.8) with depth (Fig. 4 and *SI Appendix*, Fig. S10), and show that the maximum magnitude of earthquakes decreases until the base of the seismogenic zone, consistent with the size of frictional “patches” decreasing with depth. Beginning near the frictional-to-viscous transition, we should also expect to see a decrease in the total seismic moment (Mo) accommodated by moderate earthquakes with depth as the megathrust slip budget increasingly becomes accommodated by viscous strain. We do indeed observe this decrease (Fig. 4), and while moderate to large earthquakes contribute to moment release outside their histogram bin in Fig. 4, this cannot explain the trend of decreasing cumulative moment with depth, because events are becoming smaller with depth. Finally, this does not prove that the aseismic component of deformation is viscous and not frictional, but it is consistent with the strength profiles.

There are several lithologies that may host earthquakes below the onset of significant viscous deformation in the metasediments. Metasomatic alteration products of metabasalts including chlorite and celadonite-rich faults are expected to persist below this transition (40, 42). At blueschist facies, glaucophane-rich rocks also potentially participate in faulting until the onset of viscous deformation (Fig. 3). In all of the subduction zones evaluated, the base of the seismogenic zone is below the mantle wedge corner. Thus, we expect that frictional deformation of serpentine, metasomatic products of the mantle wedge, may be responsible for seismicity (11, 12). This is consistent with the hypothesis of ref. 11 that entrained serpentine from the mantle wedge contributes to or dominates seismicity. We find that talc is expected to deform by viscous processes from its development at the mantle wedge, indicating that it is not likely a first-order control on the base of the seismogenic zone (*SI Appendix*, Fig. S3) (e.g., ref. 53).

Although there are clearly lithologies capable of frictional failure, it is not obvious why seismicity ceases at the base of the seismogenic zone and we propose three explanations: 1) The lithologies hosting earthquakes at these depths undergo temperature-dependent changes in rheology, either due to a transition from rate-weakening to rate-strengthening frictional behavior or due to enhanced viscous deformation. 2) As the viscous strength of the metasediments and other lithologies decreases with increasing temperature, there is insufficient stress to load heterogeneities to failure (e.g., ref. 54). 3) Finally, below the seismogenic zone, the size of heterogeneities become too small to resolve with global catalogs datasets. Thus, our determination of the base of the seismogenic zone may be limited by our observations since we are only considering earthquakes with Mw ≥ 5 in this study. Smaller earthquakes are more challenging to robustly characterize, as they require local arrays to characterize the focal mechanism and locate to the plate interface. Also, we are limited by a relatively short time period of well recorded earthquakes (since 1976 for the GCMT catalog), which may be too short to fully characterize the full seismogenic behavior of a margin. However, even if this is the case, it would be consistent with our model, which predicts that as the size of heterogeneities decreases with depth, so too should the magnitude of the megathrust earthquakes.

### Comparison to Lay et al. (1) Domains.

We find broad consistency between the depths at which metasediments can accommodate subduction by viscous deformation and the maximum depths at which earthquakes can nucleate and grow into large events, although the considerable uncertainties limit the precision of this depth range. Our proposed framework also feasibly explains the elevated high frequency content for megathrust events that rupture below this depth range (55). However, there are several aspects of our comparison to the domain segmentation defined by ref. 1 that require clarification and further evaluation.

First, the definitions of domains by ref. 1 are based on event centroid depth and short period energy release, not nucleation depth. For large earthquakes, centroid and nucleation depth can differ significantly: the rupture behavior of the 2021 Mw 8.1 Kermadec earthquake illustrates implications of these differences (56). This event was preceded by a Mw 7.4 foreshock with a centroid depth of 45 km and a hypocenter depth of 43 km, making the foreshock a Domain C event by the categorization of ref. 1 and consistent with our framework. However, the main event had a hypocenter depth of 29 km, consistent with our proposed framework, but is described as a Domain C event given the more empirical characterization of ref. 1 because the centroid depth is 40 km (56). This event is entirely consistent with our interpretation that earthquakes grow to be large when they nucleate and begin their growth within Domain B. However, it also highlights that the downdip growth of these events are complex and may also have a relationship with the MWC, which is largely controlled by the thickness of the overriding crust.

### Rheologic Uncertainty.

The onset of viscous deformation in the sediments is predicted due to dislocation creep in quartz with dissolution-precipitation creep predicted at greater depths (*SI Appendix*, Fig. S1). There is uncertainty in applying dissolution-precipitation creep flow laws to geologic conditions because they are challenging to characterize experimentally and strongly dependent on grain size; however, dislocation creep of quartz is arguably the most well-constrained geologic flow law, does not depend on grain size, and has been tested against geologic samples (57). Nevertheless, field studies consistently show that pressure solution creep is most common in metasediments along the megathrust (58, 59), and future work will likely confirm that pressure solution creep controls deformation of the metasedi- ments at shallower conditions than we estimate (e.g., ref. 10). Therefore, dislocation creep provides the maximum depth at which the transition to fully viscous deformation of sediments is expected (e.g., ref. 15), which reinforces our conclusion that the transition to predominately viscous deformation is significantly shallower than the base of the seismogenic zone.

### Relevance to Other Subduction Zones.

Although we evaluate a range of thermal gradients, the hottest subduction zones (e.g., Cascadia) are not included because there are an insufficient number of megathrust earthquake recordings along these margins for a detailed study of the seismogenic zone. Thus, we cannot evaluate whether our results are applicable under the highest geothermal gradients. Several authors show that the onset of viscous deformation is consistent with the base of the locked zone in Cascadia, which is generally inferred to represent the base of the seismogenic zone (10). However, our results indicate that this interpretation may not be straightforward, because it is not clear whether the hottest subduction zones lack depth-dependent segmentation between the onset of viscous deformation and the base of the seismogenic zone or if there are just not enough small to moderate plate interface earthquakes in the seismic recording era to draw a distinction. For instance, ref. 60 pointed out that in hot subduction zones the mantle wedge corner is below the predicted onset of viscous deformation, and thus lithologies like serpentine that may host earthquakes along the cooler margins are deforming here at higher temperatures. These conditions may favor their participation in deep slow slip events rather than earthquakes (e.g., ref. 61), which are correlated with high geothermal gradients. However, these subduction zones also exhibit extremely low seismicity rates above the frictional-to-viscous transition and the mantle wedge, indicating that the material properties above the mantle wedge may also be unfavorable for earthquake nucleation.

Although here we focused on subduction zones with siliciclastic inputs, we propose that the methods we use should be applicable as long as the thermal gradient and subducting material are constrained. We suggest a means of testing this model will be to evaluate subduction zones with differing inputs and including those with oceanic overriding plates. For instance, one could use a similar approach for understanding seismogenesis where subducting material including pelagic carbonates, bearing in mind that the rheology of carbonates is more complex than siliciclastics. However, we advise caution when comparing regions where inputs differ, and note that other settings or sediment inputs likely contribute to the variability seen by others (30, 53).

## Conclusions

We investigate controls on the depth-dependent segmentation of the megathrust seismogenic zone by comparing rheologic strength profiles to megathrust earthquake depths, including the hypocentral depths of large earthquakes and the depth extent of interface seismogenesis, for six subduction segments. From the results, we propose rheologic controls that can explain megathrust segmentation with depth.

[1] We propose that earthquakes nucleate and grow to great earthquakes at depths where all abundant lithologies fail frictionally. A frictional-to-viscous transition occurs when metasediments can accommodate subduction through viscous deformation, and this transition delineates the depth extent of large rupture nucleation. Across the subduction zones evaluated, which have predominantly pelitic inputs, this transition is predicted at 300 ± 60 ^°^C.
[2] We propose that the base of the seismogenic zone occurs deeper than this frictional-to-viscous transition due to structural and lithologic heterogeneity. Viscous shearing of metasediments loads entrained blocks of viscously stronger rocks (e.g., metamafic or metasomatic lithologies) to frictional failure, and both the size and frequency of these blocks decreases with depth until failure is no longer detectable. This seismically identifiable base of the seismogenic zone occurs at temperatures near 500 °C in the subduction zones evaluated. We propose the most likely candidate lithologies for seismic slip at these deeper depths are blocks of serpentinite, blueschist, and other metamafic rocks.

## Materials and Methods

### Earthquake Data.

To determine the location of base of the seismogenic zone geophysically, we compile earthquakes with moment tensor solutions and magnitudes ≥Mw 5.0 that occur within 75 km along strike of each vertical profile. To understand the segmentation of slip behavior, we also compile the rupture extents of Mw ≥ 7.8 earthquakes from the published literature (Table 1).

Magnitudes and moment tensors are sourced from the Global CMT Catalog for thrust-faulting events from 1978 to 2024 (49, 50), and hypocentral event locations are taken from the ISC Bulletin (51, 52). To identify megathrust events, we project the earthquakes parallel to the megathrust strike onto the vertical profile and compare their depths with slab top geometries from Slab2.0 and Slab1.0 (20, 62). Thrust events that project near to the slab top are interpreted as occurring on the megathrust and are used to define the base of the seismogenic zone (Table 1). To determine whether the aftershocks of large earthquakes occur over a distinct depth range due to higher strain rates, we evaluate earthquakes occurring 5 y after large earthquakes separately, and find no difference in the seismogenic zone (*SI Appendix*, Figs. S5–S9).

For comparison, we also provide the base of the seismogenic zone determined by ref. 28 in Table 1. Although they use a different earthquake catalog and compile over significantly larger segments, our results are consistent within 5 km.

### Strength Profiles.

To determine the depths at which frictional failure is suppressed in favor of viscous deformation of the metasediments, serpentinite, and blueschist, we create strength profiles that depend on both pressure and temperature. We also evaluate viscous deformation of talc and find that it is inconsistent with the base of the seismogenic zone (*SI Appendix*, Fig. S3). For the temperature-depth (and thus pressure) profiles, we use the thermal models from ref. 63 for each of the six segments and include uncertainties of ref. 29 (*SI Appendix*). We assume that brittle deformation occurs by frictional failure and compute the failure using a range of effective friction coefficients from 0.03 to 0.2 bracketed by thermal models and mechanical models of forearc topography (30, 64, 65) (*SI Appendix*, Fig. S1).

We investigate subduction segments where the input sediments are dominantly composed of clay-bearing siliciclastics (pelites), to evaluate the viscous rheology of metasediments we take the thickness of the metase- dimentary layer determined through seismic imaging and consider up to one order of magnitude variation to reflect potential effects of underplating or basal erosion (*SI Appendix*, Table S1). We use the approach of ref. 59 to approximate the rheology of a weak mica phase (25 % mica by volume) distributed within a load bearing framework of quartz. The strength of this system is dominated by quartz. For the quartz component, we consider both dislocation creep and dissolution-precipitation (pressure solution) creep. We use the dislocation creep flow law from ref. 57 and thin-film dissolution-precipitation creep model of ref. 66.

We also consider viscous deformation of the subducting crust (glaucophane) and mantle wedge (antigorite serpentine). There are no published flow laws for dislocation creep of glaucophane, and so we use the diffusion-like flow law for microboudinage of ref. 67 to evaluate viscous deformation. At depths below the mantle wedge where serpentine is expected to become significant, we consider low-temperature plasticity of antigorite using the flow law of ref. 68. We evaluate viscous deformation of glaucophane and serpentine over shear zone thicknesses of 50, 500, and 5,000 m in all subduction zones (Fig. 3 and *SI Appendix*, Fig. S2).

## Supplementary Material

Appendix 01 (PDF)

## Data Availability

Thermal model results, strength profiles, and earthquakes data have been deposited in Zenodo (10.5281/zenodo.20059294) (36).
