# Supplementary material for: Rheologic controls on the depth dependence of megathrust earthquakes
Source: Proc Natl Acad Sci U S A. 2026 Jun 25;123(26):e2535447123. doi: 10.1073/pnas.2535447123 (PMC13320668; doi:10.1073/pnas.2535447123)
Supplement: Supplementary file 1 — Appendix 01 (PDF) [file pnas.2535447123.sapp.pdf]

Supporting Information for

Rheologic controls on the depth-dependence of megathrust earthquakes

Melodie E. French, Jonathan R. Delph and Cailiey B. Condit

Melodie French.
E-mail: mefrench@rice.edu

This PDF file includes:

Supporting text
Figs. S1 to S10
Table S1
SI References

Supporting Information Text

1. Details of the thermal models

To create strength profiles, we create temperature profiles using the thermal models of (1). This model is an analytical approximation of temperature with depth and it agrees within several percent of numerical solutions (1). The model inputs are the subduction specific parameters: slab age, convergence rate, and an effective friction coefficient for shear heating, μ'_T (Table S1). (2) constrained μ'_T with regional heat flow data in the regions we evaluate, and we use their results. Because the (1) model uses a depth-invariant effective friction coefficient to account for shear heating, when viscous strength is much lower than frictional strength, this model may overestimate temperature. However, this is not of serious concern to us, as we are interested primarily in the depth at which viscous deformation at plate rates becomes weaker than frictional failure. Slab age and convergence rate are taken from the Submap database (3) and slab geometry is from Slab2.0 (4) (Table S1). All other parameters use are taken from (1).

2. Construction of the strength profiles

To construct strength profiles, we consider both frictional failure and viscous constitutive behavior (Figure S1). We assume that brittle deformation occurs by frictional slip and take the effective friction coefficient $\mu' = \tau/\sigma_n$, where τ is shear stress and σ_n is the total normal stress. We consider values of μ' from 0.03, as a lower bound, to 0.2, as an upper bound within an average of 0.12. The effective friction coefficient for shear heating provided by thermal models (μ'_T) is a lower bound on the frictional failure strength, as these are dynamic rather than static friction coefficients. These values are consistently as low as 0.03 (2, 5, 6). On the other hand, mechanical models of accretionary prism taper and forearc topography indicate frictional failure strengths, which is the measure of strength represented in our strength profiles, from 0.1 to 0.2 (7, 8). The shear stress at frictional failure is approximately $\tau(D) = \mu'\sigma_V(D)$ where $\sigma_V(D)$ is the vertical stress with depth (e.g., (9)). We calculate the total vertical stress with depth assuming a lithostatic gradient, and use densities of 2600 kg/m^3 for the accretionary prism and crust and 3300 kg/m^3 for the mantle.

The shear strength due to viscous creep is determined by assuming a strain rate equal to the subduction rate divided by the layer thickness (Table S1) (Figure S1). For the metasediments, we assume that the thickness of the layer is equal to that determined through seismic imaging (Table S1), and also evaluate viscous deformation assuming sediments are thinned or thickened one order of magnitude to consider the potential effects of layer thinning or thickening (Figure S2). For glaucophane and antigorite-rich serpentinite, we consider deforming layers 50, 500, and 500 m thick (Figure S2). We then solve the rheologic flow laws for differential stress, σ_d and calculate the shear stress as $\tau = \sigma_d/\sqrt{3}$. We relate experimental axial strain-rate, $\dot{epsilon}$ to engineering shear strain-rate, $\dot{\gamma}$, as $\dot{\epsilon} = \dot{\gamma}/\sqrt{3}$ (10).

A. Metasediments. We investigate subduction segments where the input sediments are dominantly composed of clay-bearing siliclastics (pelites). To evaluate the viscous rheology of metasediments, we use the approach of (11), where the strain rate assumes a layer thickness equivalent to the regionally-constrained subducted sediment thickness (Table S1), and also evaluate thicknesses one order of magnitude greater and less than this (Figure S2). We use this model to approximate the rheology of a weak mica phase (25 % mica by volume) distributed within a load bearing framework of quartz. The resulting viscous strength is $\tau_s = 0.25\tau_m + 0.75\tau_q$ (12), where tau_m is the strength of mica and τ_q is the strength of quartz. Phyllosilicate deformation is predominantly by glide parallel to the basal planes at these conditions ($\tau_m = \tau_{pl}$), and we use the flow law of (13), who re-fit the muscovite glide data of (14) accounting for the σ_d^2 dependence that is necessary for accurate extrapolation to low strain rates and stresses (Equation 1).

$$\dot{\epsilon} = A_{mg}\sigma_d^2 \exp(a_{mg}\sigma_d - Q_{mg}/(RT)) \qquad [1]$$

where A_{mg} is 1×10^{-12} s^{-1}, a_{mg} is 0.5 MPa^{-1}, and Q_{mg} is 47 kJ/mol (14).

For the quartz component, we consider both dislocation creep and dissolution-precipitation (pressure solution) creep. Dislocation creep follows the well-established flow law from (15):

$$\dot{\epsilon} = 10^{11.2} f_{H_2O}(P,T)\sigma_d^4 \exp(-Q/RT) \qquad [2]$$

where $Q = 135$ kJ/mol and $f_{H_2O}(P,T)$ is the water fugacity, calculated from pressure and temperature using (16), assuming lithostatic fluid pressure.

For dissolution-precipitation creep of quartz, we evaluate use the thin-film model, whereby diffusion occurs through structured water films along grain boundaries. The constitutive behavior is given by:

$$\dot{\epsilon} = \frac{A_R V_m \rho_f c(T) D(T) w \sigma_d}{\rho_s R T d^3} \qquad [3]$$

where $A_R = 32$ is a geometric constant for tabular grains, V_m is the molar volume of quartz (17), ρ_f is the fluid density as a function of fluid pressure and temperature (18), ρ_s is the solid density (2600 kg/m^3 for quartz), $c(T, P_f)$ is mineral solubility as a function of temperature as function of fluid pressure and temperature (19), $D_(T)$ is the diffusivity of grain boundary thin films as a function of temperature, w is the thin film width in meters, R is the gas constant, d is grain size in meters, and T is temperature. For $D(T)$, we use the bulk aggregate Si diffusivity in novaculite of (20) ($D_{bulk} = 3.7 \times 10^{-10} \exp(-137kJ/mol/(RT))$),

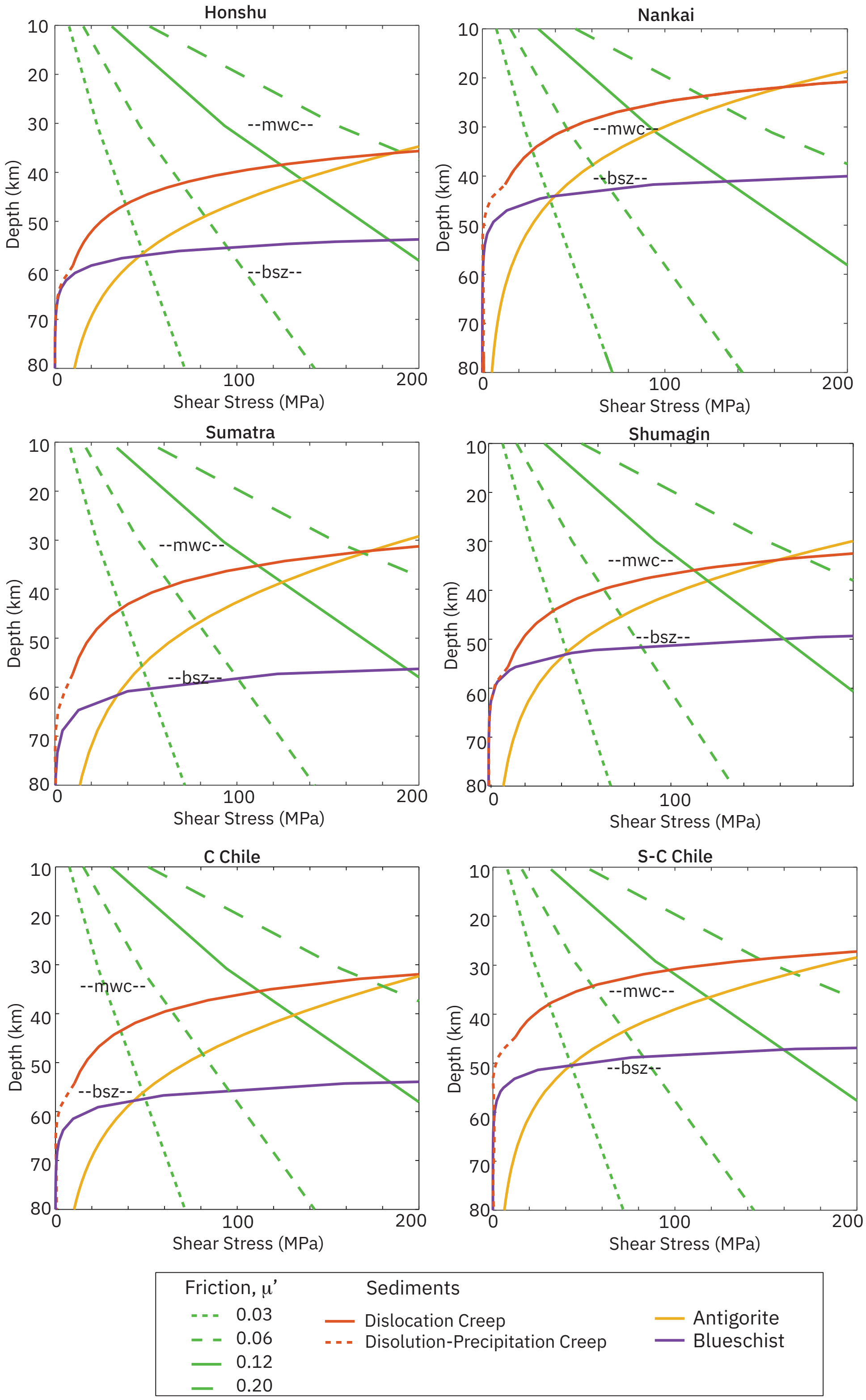

Fig. S1. Strength profiles for the six subduction segments showing frictional failure strengths for different values of effective friction coefficient (μ') across the range we evaluated (0.03 to 0.2). Viscous flow strength for metasediments, antigorite-rich serpentinite, and blueschist are shown for subduction rates. Metasediment layer thickness is given in Table S1 and serpentinite and blueschist are both assumed 500 m thick in this plot. The depth ranges where quartz is predicted to deform by dislocation creep versus dissolution-solution creep are indicated.

assume that diffusivity through the grains is a negligible contribution to bulk diffusivity (as they show), and multiply by the ratio of grain size to grain boundary thickness in their experiments (600) to get grain boundary diffusivity of $D_{gb} = 2.2 \times 10^{-7} \exp(-137kJ/mol/(RT))$ (20). We approximate a grain boundary width of $w = 150$ nm, because the grain boundary width for silicate-rich rocks has been estimated as 5 nm (20, 21) and (22) showed that phyllosilicates along grain boundaries increase the effective width by a factor of 30.

Applying the microphysical mixing model that includes glide of the mica phase reduces the strength of the schist relative to pure quartz slightly for all dislocation and dissolution-precipitation creep models. This rheologic model results in a transition from deformation dominated by dislocation creep of quartz in the schist at lower temperatures, higher stresses, and shallower depths to dissolution-precipitation creep at higher temperatures, lower stresses, and greater depths (Figure S1). (13) show that the (12) model overestimates the composite flow strength of quartz-mica aggregates in deformation experiments, and thus we consider the mixing relationship we use to provide an upper bound in mica-bearing metasediment strength.

B. Serpentinite. At low deformation rates antigorite deforms by low temperature plasticity in the basal plane (23). The constitutive equation for low-temperature plasticity of antigorite is:

$$\dot{\epsilon} = exp{-0.624}(\frac{\sigma_d}{35 \times 10^9 Pa})^2 exp\frac{-86.3 \times 10^3}{RT}(1 - (\frac{\sigma_d}{2.42 \times 10^9 Pa}))^{1.18} \quad [4]$$

To evaluate the range of feasible strain rates, we assume that the total plate convergence rate is accommodated over zones 50, 500, and 5000 m thick which spans geologic observations (24, 25). Although dissolution-precipitation of serpentine has been documented in the rock record, its rheology is not established and low-temperature plasticity therefore represents an upper bound on the viscous strength of antigorite (26).

C. Mafic Blueschist. Glaucophane is produced during blueschist-facies metamorphism of basalt. Field studies show that glaucophane deforms by dislocation-mediated processes and dynamic recrystallization beginning at ∼350°C (27–30), but there are no published flow laws. To evaluate viscous deformation, we use the diffusion creep law for glaucophane of (31) given by:

$$\dot{\epsilon} = 2.43 \times 10^{11} \sigma_d^2/\delta \exp(-Q/RT) \quad [5]$$

where $Q = 384$ kJ/mol and $\delta = 0.5$ μm. To evaluate the range of feasible strain rates, we assume that the total plate convergence rate is accommodated over zones 50, 500, and 5000 m thick zones, which span geologic observations (28, 32, 33).

D. Talc. At low deformation rates talc deforms by low temperature plasticity in the basal plane (34). We evaluate shear strain-rates assuming thickness may range from 5 to 500 m, encompassing geologic observations (Figure S3) (24, 35). The constitutive equation for low-temperature plasticity of talc is:

$$\dot{\gamma} = (\exp 8.55)(\frac{\tau}{22 \times 10^9 Pa})^2 \exp\frac{-120 \times 10^3}{RT}(1 - (\frac{\tau}{0.3 \times 10^9 Pa}))^{1.5} \quad [6]$$

Talc is most likely to be produced in the mantle wedge of the subduction zones investigated (e.g., Easthouse, Hoover et al., 2025), and we find that talc low-temperature plasticity is expected to occur from its development, which is significantly above the base of the seismogenic zone in all cases investigated.

3. Uncertainty

Uncertainties in the depths at which lithologies can accommodate aseismic creep at subduction rates arise from uncertainties in the thermal gradients, the frictional failure strength, rheologic flow laws, and layer thicknesses. We describe constraints on these uncertainties below, and then how they are propagated into an uncertainty in the frictional-to-viscous transition.

The analytical thermal models allow for the quantification of uncertainties, which (2) and (6) show range from 20-40%. These large uncertainties are dominated by the epistemic uncertainties in surface heat flux, depth to the plate interface, and radiogenic heat production. As discussed in Section 2, frictional failure strength is another epistemic uncertainty in determining the frictional-to-viscous transition, which evidence indicates lies between 0.03 and 0.2. Higher values of frictional failure result in shallower frictional-to-viscous transitions.

Determinations of viscous flow strengths at subduction rates suffer from uncertainty in both the rheologic flow laws and the thicknesses of deforming layers. To address the latter point, we consider three orders of magnitude variation in layer thickness constrained by geologic observations (36, 37). Even considering this potential variation, the metasediments are expected to undergo viscous creep of the interface at the shallowest depth and thereby control the frictional-to-viscous transition (Figure S2).

There is considerable uncertainty in our current understanding of the viscous flow strengths of some of the lithologies and deformation mechanisms. However, the for the purposes of this paper in which we aim to demonstrate that viscous flow can explain segmentation of slip behavior, we have chosen flow laws and parameters that provide upper bounds on strength, and therefore represent maximum frictional-to-viscous transition depths. For the metasediments, the uncertainties arise from choices mica content, quartz-mica mixing relations, the pressure solution creep flow law, and random errors in flow law parameters like activation energy. The last of these is negligible compared to the others. We used a quartz-mica mixing relation that provides an upper bound on mixture strength compared to laboratory data (e.g., (13)), and the quantity of mica (25 %) is comparable

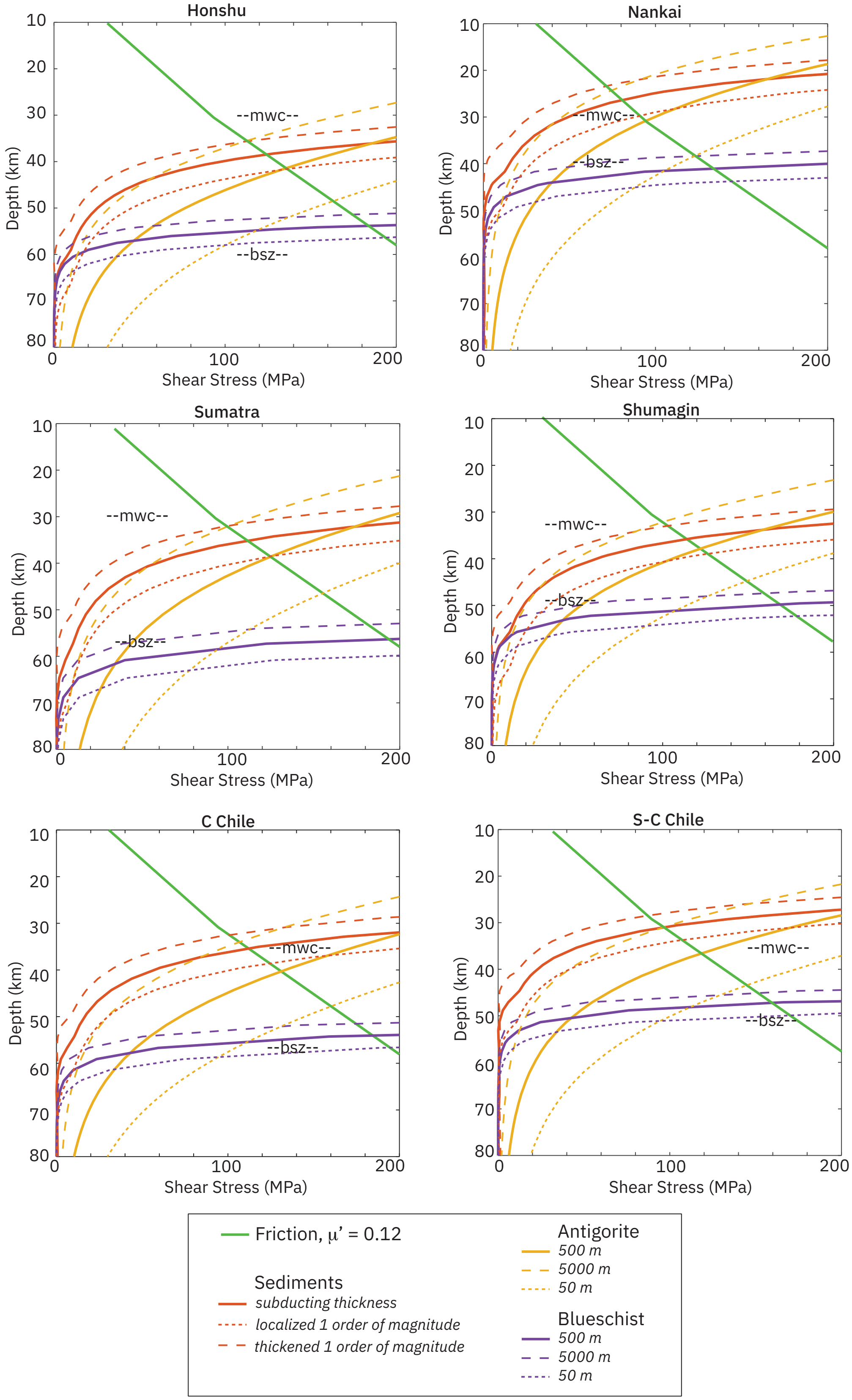

Fig. S2. Strength profiles showing the influence of deforming layer thickness on the onset of viscous deformation in the metasediments, antigorite, and blueschist. The viscous strength of sediments is shown for strain-rates determined from geophysical imaging of subducting sediment thickness (Table S1), as well as ±1 order of magnitude of this thickness. Viscous strength of antigorite and blueschist are shown for 50 m, 500 m, and 5000 m layers.

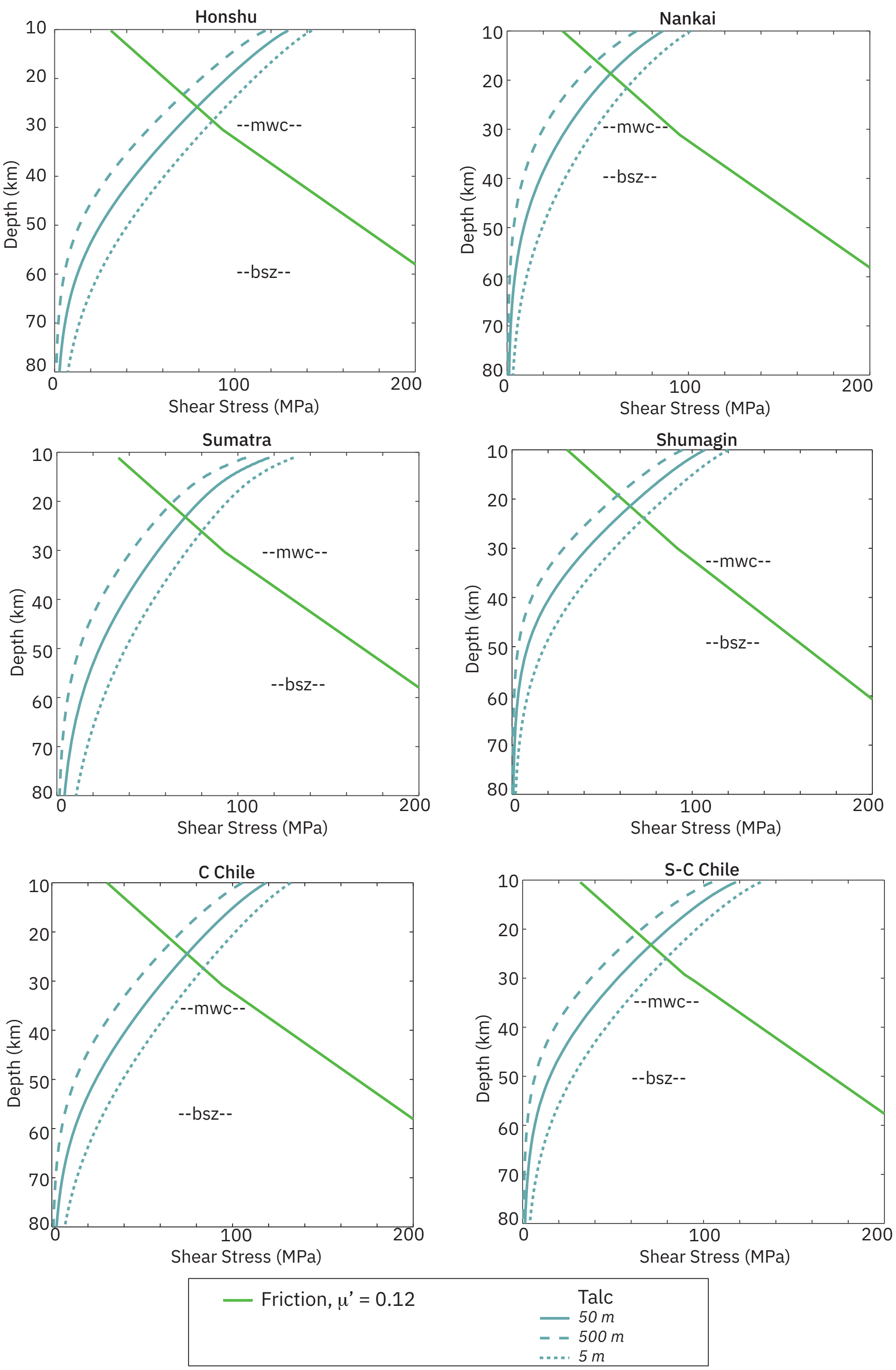

Fig. S3. Strength profiles for talc low-temperature plasticity showing the influence of deforming layer thickness for a range of geologically feasible thicknesses (5, 50, 500 m). The onset of low-temperature plasticity is expected as soon as talc develops in the mantle wedge corner (mwc) and well above the base of the seismogenic zone (bsz).

to field observations and a median value of that determined using petrologic models (11, 38). Decreasing this value to 10 %, which is the minimum value of (38) would have negligible effect on these results. By far the greatest uncertainty lies in the pressure solution creep flow law. Different formulations of this flow law that allow for differences in diffusion pathway structure and fluid diffusivity can result in up to 13 orders of magnitude strain rate for a given stress (20). We have used the most conservative of these which results in sediment deformation by dislocation creep at shallower depths than pressure solution creep becomes significant (Figure S1). This is contrary to field evidence that shows predominately pressure solution creep at conditions that our results predict dislocation creep (11, 36, 39). Thus, our choice of rheology again provides a maximum depth at which we expect viscous creep of sediments to accommodate subduction.

We use Monte Carlo simulations to evaluate uncertainty in the frictional-to-viscous transition. We run 10^5 simulations where thermal gradients , effective friction coefficient, and sediment thickness vary within range of their uncertainties. To consider the maximum uncertainty in thermal gradients, for each margin we consider a normal distribution centered on the thermal model outputs from parameters in Table S1 truncated at ±40% variability according to (6). The frictional failure strength follows a uniform distribution between μ' of 0.03 and 0.2, resulting in an average value of 0.12. Metasediment thicknesses follow a log-normal distribution centered at the values in Table S1 and truncated at ±1 order of magnitude difference in thickness. The results of the simulations show a skew towards greater depths (Figure S4), which is due to the non-linear effect of lower frictional failure stresses on the frictional-to-viscous transition (Figure S1). Accordingly, we report the median result and the range of values between the 16-84% percentiles, which represent 1 standard deviation for a symmetric distribution (Figure S4).

Table S1. Subduction Profile Parameters

Subduction segment	V_c [a] mm/yr	V_n mm/yr	t_0 Myr	μ'_T	μ'_T(range)	T_s [b] m
Honshu	92	89	130	0.06	0.03-0.09	200
Nankai	58	44	17	0.06	0.02-0.11	700
Sumatra	42	41	50	0.06	0.02-0.09	500
Shumagin	66	63	55	0.06	0.03-0.09	150
South-Central Chile	74	55	34	0.06	0.03-0.09	2500
Central Chile	74	70	40	0.07	0.03-0.11	900

[a] V_c, V_n, t_0, and μ' are parameters in the thermal model of (1). V_c is plate convergence velocity, V_n is trench-normal component of convergence velocity, t_0 is oceanic plate age at the trench, and μ' is the effective friction coefficient. V, V_n, and t_0 are from the Submap database at https://submap (3). μ'_T are the regional values of the shear heating effective friction coefficient determined by (2), and μ'_T (range) is the range of possible values that encompass uncertainty in this parameter. Where the range is evaluated directly by (2), it is shown, otherwise a range of ±50% is assumed. [b] T_s is subducting sediment thickness from (49) (Japan trench), (50) (Sumatra), (51) (Shumagin), (52, 53) (central Chile), and (54) (south-central Chile)

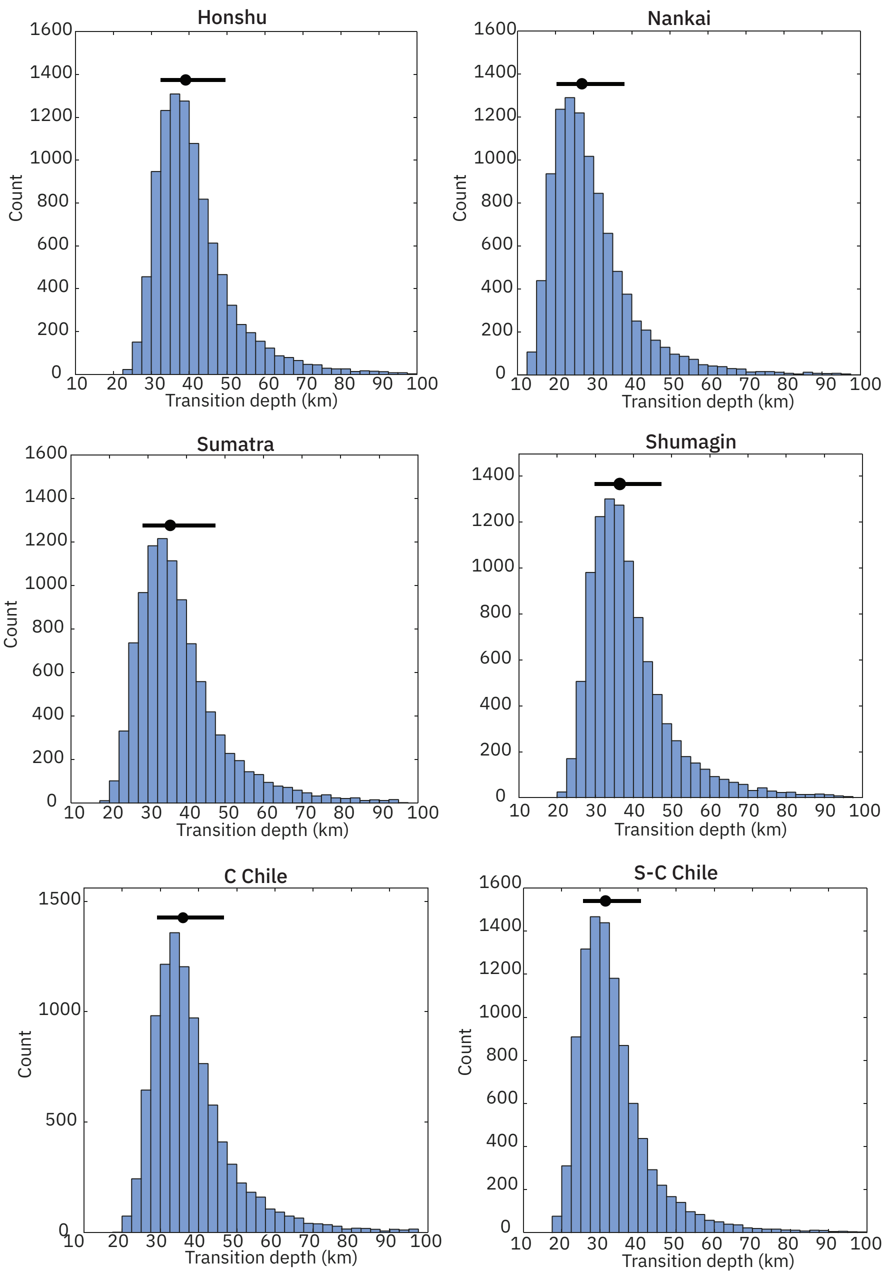

Fig. S4. The results of Monte Carlo simulations for the depth at which viscous flow of metasediments can accommodate subduction at lower shear stress than frictional failure, resulting in a frictional-to-viscous transition. Histograms show the results of 10^5 simulations that include variations in thermal gradient, frictional failure strength, and metasediment thickness that encompass uncertainties in these parameters. Black dots and bars show the median results and 16-84 % range, respectively.

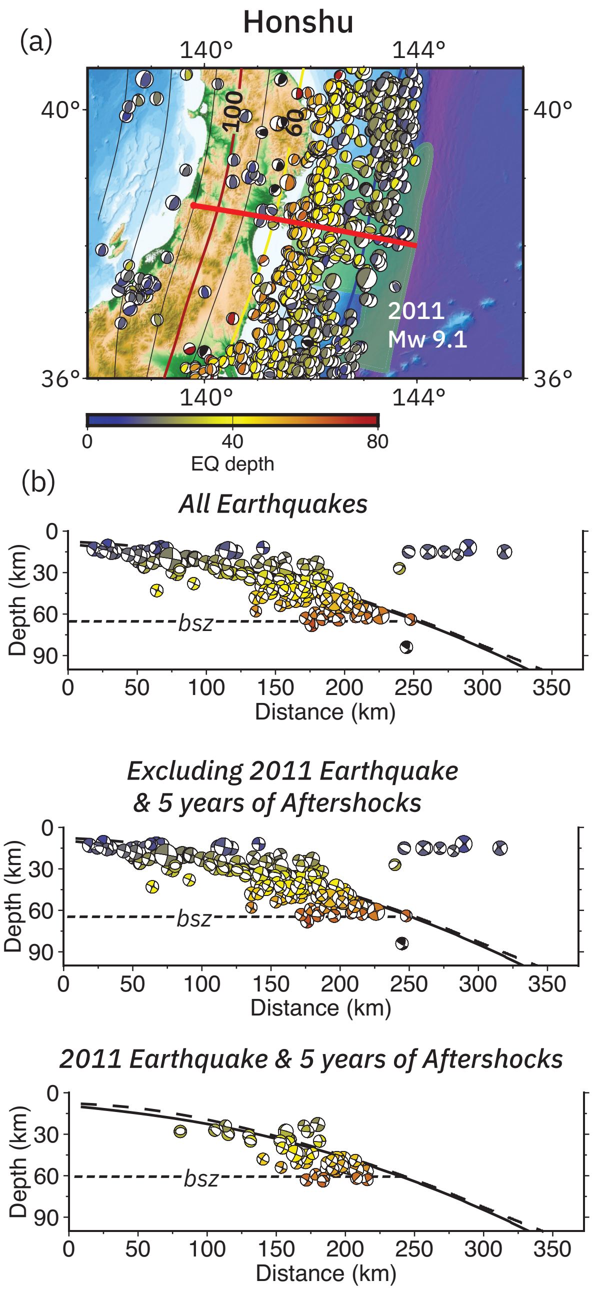

Fig. S5. (a) Map and topography showing the location of the Honshu, Japan profile in red. Slab contours are shown in 20 km intervals and are from Slab 2.0 (4). Focal mechanisms from thrust events are shown and color coded according to depth. The slip area of the 2011 Mw 9.1 earthquake is shown (40). (b) Thrust events within 75 km of the profile are projected parallel to strike onto the profile. The Slab2.0 (solid) and Slab1.0 (dashed) slab interfaces are shown (4, 41). Events that project to the slab top are used to identify the base of the seismogenic zone (bsz). The 2011 Mw 9.1 earthquake and five years of its aftershocks are shown separately from the rest of events to demonstrate that post-seismic creep does not influence identification of the seismogenic zone.

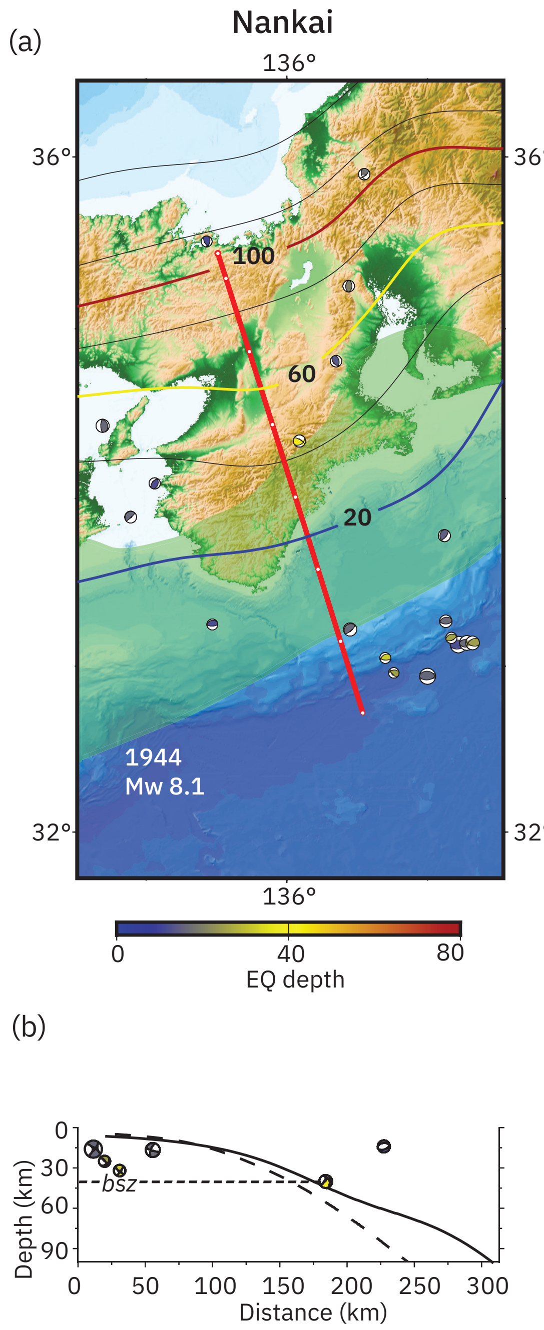

Fig. S6. (a) Map and topography showing the location of the Kii peninsula profile in red. Slab contours are shown in 20 km intervals and are from Slab2.0 (4). Focal mechanisms from thrust events are shown and color coded according to depth. Approximate coseismic slip region is shown for the 1944 Mw 8.8 earthquake (42). (b) Thrust events within 75 km of the Kii profile are projected onto the profile to identify the base of the seismogenic zone (bsz). Because there is little seismicity, we identify the minimum depth of the bsz. The Slab2.0 (solid) and Slab1.0 (dashed) slab interfaces are shown (4, 41).

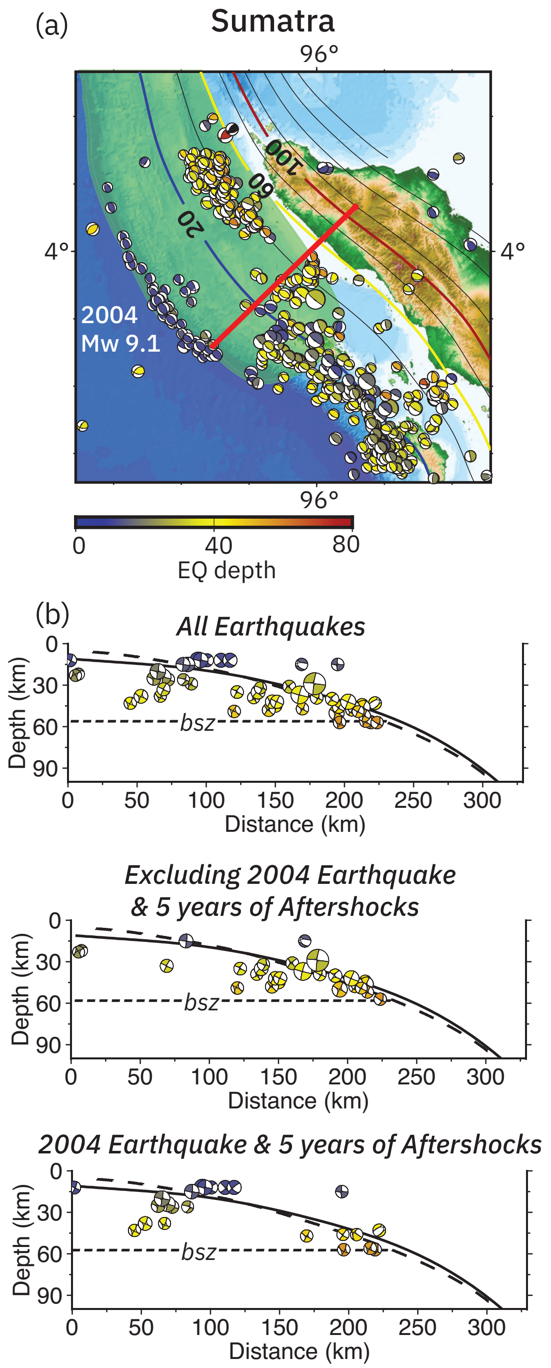

Fig. S7. (a) Map and topography showing the location of the Sumatra profile in red. Slab contours are shown in 20 km intervals and are from Slab2.0 (4). Focal mechanisms from thrust events are shown and color coded according to depth. The rupture boundary is shown for the 2004 Mw 9.1 earthquake (43, 44). (b) Thrust events within 75 km are projected onto the profile along strike. The Slab2.0 (solid) and Slab1.0 (dashed) slab interfaces are shown (4, 41). Events that project to the slab top are used to identify the base of the seismogenic zone (bsz). The 2004 Mw 9.1 earthquake and five years of its aftershocks are shown separately from the rest of events to demonstrate that post-seismic creep does not influence identification of the seismogenic zone.

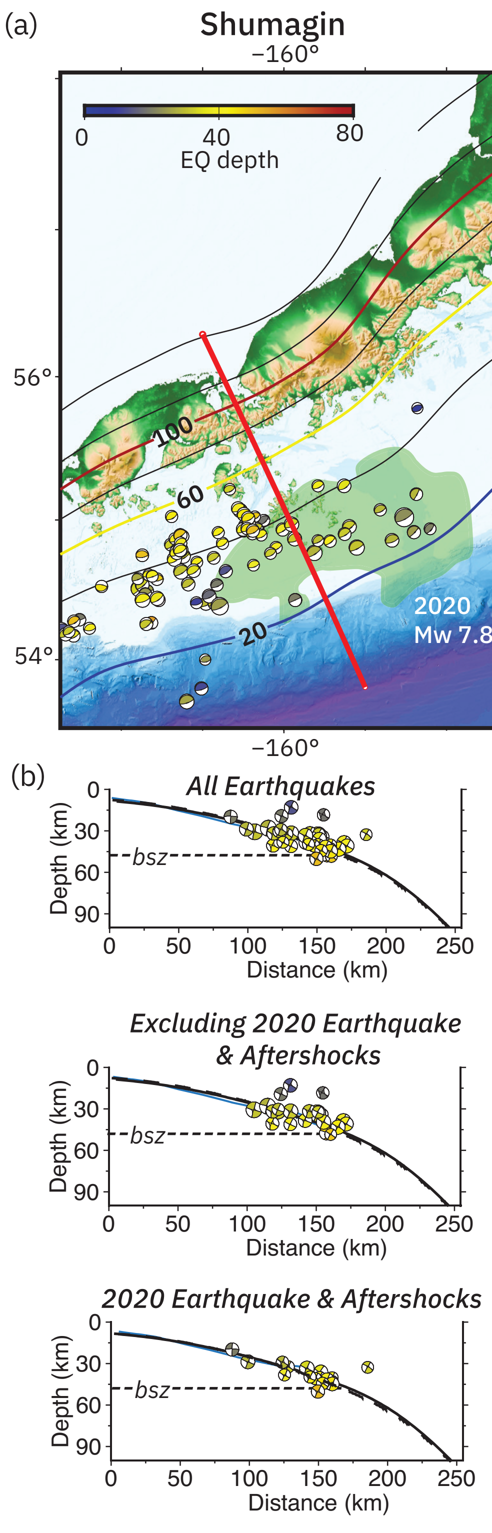

Fig. S8. (a) Map and topography showing the location of the Alaska peninsula Shumagin profile in red. Slab contours are shown in 20 km intervals and are from Slab 2.0 (4). Focal mechanisms from thrust events are shown and color coded according to depth. The slip region is shown for the 2020 Mw 7.8 earthquake (45). (b) Thrust events within 75 km of the profile are projected along strike onto the profile. The Slab2.0 (solid) and Slab1.0 (dashed) slab interfaces are shown (4, 41) along with the inferred plate boundary from Line 5 of (46) (blue). Events that project to the slab top are used to identify the base of the seismogenic zone (bsz). The 2020 Mw 7.8 and subsequent earthquakes are shown separately from the rest of events to demonstrate that post-seismic creep does not influence identification of the seismogenic zone.

 Melodie E. French, Jonathan R. Delph and Cailiey B. Condit

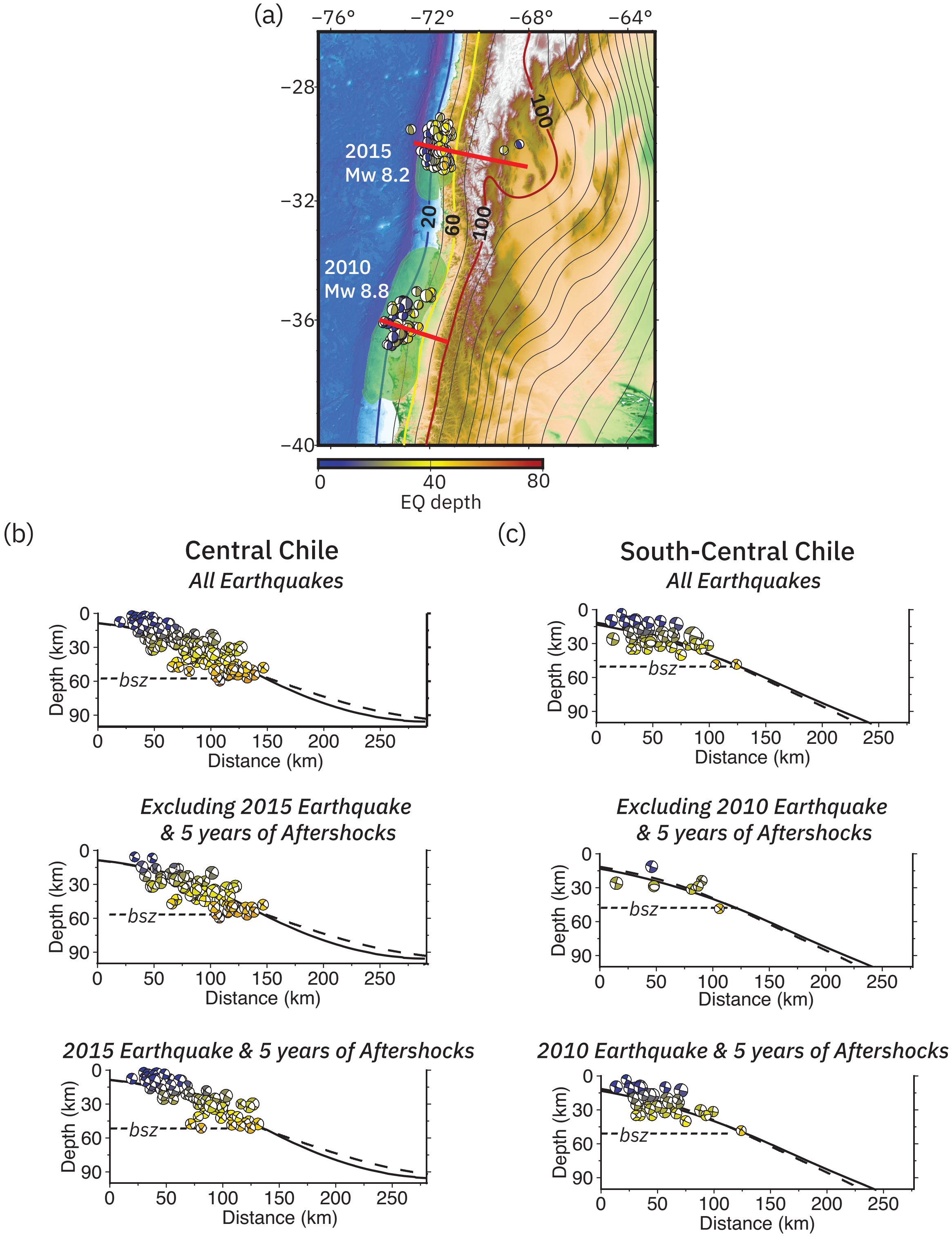

Fig. S9. (a) Map and topography showing the locations of the central and south-central Chile profiles in red lines. Slab contours are shown in 20 km intervals and are from Slab 2.0 (4). Focal mechanisms from thrust events are shown and color coded according to depth. The slip areas are shown for the 2010 Mw 8.8 Maule earthquake (47) and the 2015 Mw 8.0 Illapel earthquake (48). Thrust events within 75 km of (b) the south-central Chile profile and (c) the central Chile profile are projected onto the profile to determine the base of the seismogenic zone (bsz). The Slab2.0 (solid) and Slab1.0 (dashed) slab interfaces are shown (4, 41). The 2010 and 2015 earthquakes and five years of their aftershocks are shown separately from the rest of events to demonstrate that post-seismic creep does not influence identification of the seismogenic zone.

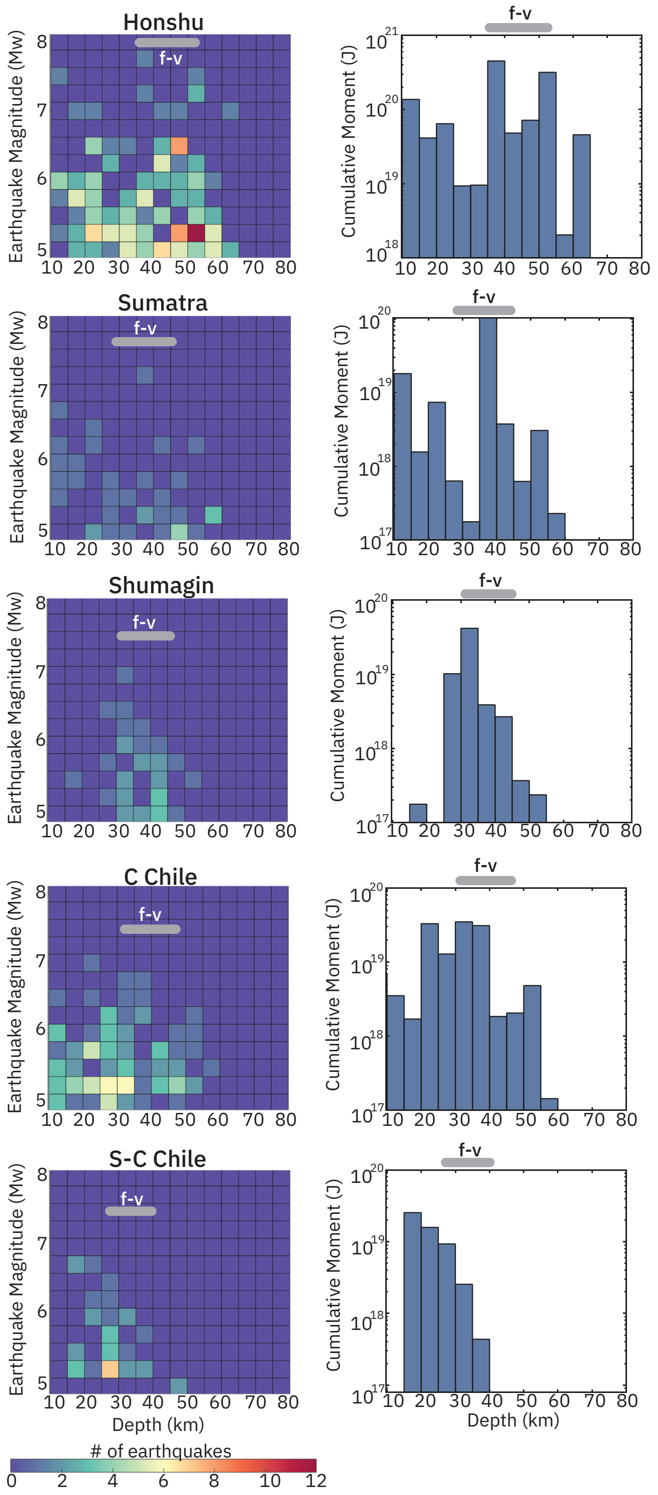

Fig. S10. (left) Heat maps showing the number of megathrust earthquakes of given magnitudes (Mw) and depths. (right) Histograms show the sum of the seismic moments accommodated by earthquakes (cumulative seismic moment, Mo) with nucleation depth. Earthquakes are thrust events within 75 km of each transect along strike and within 12 km orthogonal distance to the slab. The frictional-to-viscous transition is indicated as 'f-v'. Earthquakes greater than Mw 7.8 are not included. Although more earthquakes are included compared to Figure 4 which includes only earthquakes within 7 km orthogonal distance, the trends are the same.

References

1. PC England, DA May, The global range of temperatures on convergent plate interfaces. *Geochem. Geophys. Geosystems* **22**, e2021GC009849 (2021).
2. PC England, On shear stresses, temperatures, and the maximum magnitudes of earthquakes at convergent plate boundaries. *J. Geophys. Res. Solid Earth* **123**, 7165–7202 (2018).
3. S Lallemand, A Heuret, Subduction zones parameters in *Reference Module in Earth Systems and Environmental Sciences.* (Elsevier), (2017).
4. G Hayes, Slab2 - A comprehensive subduction zone geometry model (2018).
5. X Gao, K Wang, Strength of stick-slip and creeping subduction megathrusts from heat flow observations. *Science* **345**, 1038–1041 (2014).
6. P England, T Matsumoto, S Wallis, Implications of surface heat flux for shear stress and temperature on the plate interface beneath northern Honshu. *Geochem. Geophys. Geosystems* **25** (2024).
7. F Dahlen, Critical taper model of fold-and-thrust belts and accretionary wedges. *Annu. Rev. Earth Planet. Sci.* **18**, 55–99 (1990).
8. R Cattin, H Lyon-Caen, J Chéry, Quantification of interplate coupling in subduction zones and forearc topography. *Geophys. Res. Lett.* **24**, 1563–1566 (1997).
9. X Gao, K Wang, Rheological separation of the megathrust seismogenic zone and episodic tremor and slip. *Nature* **543** (2017).
10. M Paterson, D Olgaard, Rock deformation tests to large shear strains in torsion. *J. Struct. Geol.* **22**, 1341 – 1358 (2000).
11. CB Condit, et al., Rheology of metasedimentary rocks at the base of the subduction seismogenic zone. *Geochem. Geophys. Geosystems* **23**, e2021GC010194 (2022).
12. MR Handy, Flow laws for rocks containing two non-linear viscous phases: a phenomenological approach. *J. structural Geol.* **16**, 287–301 (1994).
13. L Tokle, G Hirth, H Stünitz, The effect of muscovite on the microstructural evolution and rheology of quartzite in general shear. *J. Struct. Geol.* **169**, 104835 (2023).
14. V Mares, A Kronenberg, Experimental deformation of muscovite. *J. Struct. Geol.* **15**, 1061 – 1075 (1993).
15. G Hirth, C Teyssier, JW Dunlap, An evaluation of quartzite flow laws based on comparisons between experimentally and naturally deformed rocks. *Int. J. Earth Sci.* **90**, 77–87 (2001).
16. KS Pitzer, SM Sterner, Equations of state valid continuously from zero to extreme pressures for H2O and CO2. *The J. Chem. Phys.* **101**, 3111–3116 (1994).
17. C Frondel, CS Hurlbut, Determination of the atomic weight of silicon by physical measurements on quartz. *The J. Chem. Phys.* **23**, 1215–1219 (1955).
18. CW Burnham, JR Holloway, NF Davis, Thermodynamic Properties of Water to 1,000°C and 10,000 Bars in *Thermodynamic Properties of Water to 1,000° C and 10,000 Bars.* (Geological Society of America), (1969).
19. RO Fournier, RW Potter, An equation correlating the solubility of quartz in water from 25° to 900°C at pressures up to 10,000 bars. *Geochimica et Cosmochimica Acta* **46**, 1969 – 1973 (1982).
20. J Farver, R Yund, Silicon diffusion in a natural quartz aggregate: Constraints on solution-transfer diffusion creep. *Tectonophysics* **325**, 193 – 205 (2000).
21. DL Ricoult, DL Kohlstedt, Structural width of low-angle grain boundaries in olivine. *Phys. Chem. Miner.* **9**, 133–138 (1983).
22. SH Hickman, B Evans, Kinetics of pressure solution at halite-silica interfaces and intergranular clay films. *J. Geophys. Res. Solid Earth* **100**, 13113–13132 (1995).
23. E Burdette, G Hirth, Creep rheology of antigorite: Experiments at subduction zone conditions. *J. Geophys. Res. Solid Earth* **127** (2022).
24. ME French, CB Condit, Slip partitioning along an idealized subduction plate boundary at deep slow slip conditions. *Earth Planet. Sci. Lett.* **528**, 115828 (2019).
25. CJ Tulley, et al., Rheology of naturally deformed antigorite serpentinite: Strain and strain-rate dependence at mantle-wedge conditions. *Geophys. Res. Lett.* **49** (2022).
26. E Amiguet, BVD MoortÃ¨le, P Cordier, N Hilairet, B Reynard, Deformation mechanisms and rheology of serpentines in experiments and in nature. *J. Geophys. Res. Solid Earth* **119**, 4640–4655 (2014).
27. B Reynard, P Gillet, C Willaime, Deformation mechanisms in naturally deformed glaucophanes: A TEM and HREM study. *Eur. J. Mineral.* **1**, 611–624 (1989).
28. Y Cao, H Jung, S Song, Microstructures and petro-fabrics of lawsonite blueschist in the North Qilian suture zone, NW china: Implications for seismic anisotropy of subducting oceanic crust. *Tectonophysics* **628**, 140–157 (2014).
29. D Kim, I Katayama, K Michibayashi, T Tsujimori, Rheological contrast between glaucophane and lawsonite in naturally deformed blueschist from Diablo Range, California. *Island Arc* **22**, 63–73 (2013).
30. JN Ott, CB Condit, M Pec, B Journaux, Dislocation creep of glaucophane in mafic blueschists during subduction: Weighted burgers vector analysis from the Catalina Schist (California, USA). *Geology* (year?).
31. L Tokle, LJ Hufford, WM Behr, LFG Morales, C Madonna, Diffusion creep of sodic amphibole-bearing blueschist limited by microboudinage. *J. Geophys. Res. Solid Earth* **128** (2023).
32. D Kim, I Katayama, K Michibayashi, T Tsujimori, Deformation fabrics of natural blueschists and implications for seismic

anisotropy in subducting oceanic crust. *Phys. Earth Planet. Interiors* **222**, 8–21 (2013).
33. S Choi, et al., Deformation microstructures of blueschists in Alpine Corsica, France, and implications for seismic anisotropy and the low-velocity layer in subducting oceanic crust. *Tectonophysics* **877**, 230297 (2024).
34. HS Shabtian, G Hirth, Creep of talc at subduction zone conditions: Implications for slow slip and strength of the lithosphere. *Geophys. Res. Lett.* **52** (2025).
35. WF Hoover, CB Condit, PC Lindquist, AC Moser, VE Guevara, Episodic slow slip hosted by talc-bearing metasomatic rocks: High strain rates and stress amplification in a chemically reacting shear zone. *Geophys. Res. Lett.* **49** (2022).
36. WM Behr, JP Platt, Rheological evolution of a Mediterranean subduction complex. *J. Struct. Geol.* **54**, 136 – 155 (2013).
37. CM Tewksbury-Christle, WM Behr, MA Helper, Tracking deep sediment underplating in a fossil subduction margin: Implications for interface rheology and mass and volatile recycling. *Geochem. Geophys. Geosystems* **22** (2021).
38. AJ Smye, PC England, Metamorphism and deformation on subduction interfaces: 2. Petrological and tectonic implications. *Geochem. Geophys. Geosystems* **24** (2023).
39. DM Fisher, G Hirth, A pressure solution flow law for the seismogenic zone: Application to Cascadia. *Sci. Adv.* **10** (2024).
40. Y Yamazaki, KF Cheung, T Lay, A self-consistent fault slip model for the 2011 Tohoku earthquake and tsunami. *J. Geophys. Res. Solid Earth* **123**, 1435–1458 (2018).
41. GP Hayes, DJ Wald, RL Johnson, Slab1.0: A three-dimensional model of global subduction zone geometries. *J. Geophys. Res. Solid Earth* **117** (2012).
42. EM Sherrill, KM Johnson, New insights into the slip budget at Nankai: An iterative approach to estimate coseismic slip and afterslip. *J. Geophys. Res. Solid Earth* **126** (2021).
43. CJ Ammon, et al., Rupture process of the 2004 Sumatra-Andaman earthquake. *Science* **308**, 1133–1139 (2005).
44. M Chlieh, et al., Coseismic slip and afterslip of the great Mw 9.15 Sumatra–Andaman earthquake of 2004. *Bull. Seismol. Soc. Am.* **97**, S152–S173 (2007).
45. C Liu, T Lay, X Xiong, Y Wen, Rupture of the 2020 Mw 7.8 earthquake in the Shumagin Gap inferred from seismic and geodetic observations. *Geophys. Res. Lett.* **47** (2020).
46. DJ Shillington, A Bécel, MR Nedimović, Upper plate structure and megathrust properties in the Shumagin Gap near the July 2020 M7.8 Simeonof event. *Geophys. Res. Lett.* **49** (2022).
47. M Moreno, et al., Toward understanding tectonic control on the Mw 8.8 2010 Maule Chile earthquake. *Earth Planet. Sci. Lett.* **321–322**, 152–165 (2012).
48. MN Shrivastava, et al., Coseismic slip and afterslip of the 2015 Mw 8.3 Illapel (Chile) earthquake determined from continuous GPS data. *Geophys. Res. Lett.* **43** (2016).
49. Y Nakamura, et al., Incoming plate structure at the Japan Trench subduction zone revealed in densely spaced reflection seismic profiles. *Prog. Earth Planet. Sci.* **10** (2023).
50. SM Dean, et al., Contrasting décollement and prism properties over the Sumatra 2004–2005 earthquake rupture boundary. *Science* **329**, 207–210 (2010).
51. J Li, et al., Connections between subducted sediment, pore-fluid pressure, and earthquake behavior along the Alaska megathrust. *Geology* **46**, 299–302 (2018).
52. R von Huene, et al., Tectonic control of the subducting Juan Fernández Ridge on the Andean margin near Valparaiso, Chile. *Tectonics* **16**, 474–488 (1997).
53. T Seno, Subducted sediment thickness and M9 earthquakes. *J. Geophys. Res. Solid Earth* **122**, 470–491 (2017).
54. KM Olsen, et al., Thick, strong sediment subduction along south-central Chile and its role in great earthquakes. *Earth Planet. Sci. Lett.* **538**, 116195 (2020).
